# Draft genome sequence of *Vibrio diabolicus* isolated from the starlet sea anemone *Nematostella vectensis*

**DOI:** 10.1128/mra.01075-23

**Published:** 2024-03-08

**Authors:** Quinton Krueger, Adam Reitzel

**Affiliations:** 1 Department of Biological Sciences, University of North Carolina at Charlotte, Charlotte, North Carolina, USA; Montana State University, Bozeman, Montana, USA

**Keywords:** *Vibrio*, host–cell interactions, marine microbiology

## Abstract

*Nematostella vectensis* has grown as a model organism for investigating host–bacteria interactions. Here, we report the full genome of *Vibrio diabolicus* NVE-VD1, an isolate from *N. vectensis* from the South Carolina Baruch Estuarine Reserve.

## ANNOUNCEMENT

*Nematostella vectensis* was collected from tidally restricted pools on Goat Island located in the South Carolina Baruch Estuarine Reserve in June 2018 (33°19′50.6″N 79°12′04.2″W). Anemones were then transported to the University of North Carolina at Charlotte (Charlotte, NC, USA). Homogenized anemone was serially diluted and plated on marine broth (24.72 g L^−1^ NaCl, 0.78 g L^−1^ KCl, 1.36 g L^−1^ CaCl_2_, 4.66 g L^−1^ MgCl_2_, 6.30 g L^−1^ MgSO_4_, 0.36 g L^−1^ NaHCO_3_, 10 g L^−1^ peptone, 3 g L^−1^ yeast extract, and 15 g L^−1^ agar) plates. A distinct colony was isolated and struck at minimum three times on fresh media to ensure a pure isolate. The isolate was then grown in marine broth and pelleted by centrifugation for DNA extraction and sequencing at Omega Bioservices. DNA was extracted with the Mag-Bind Universal Pathogen DNA Kit (Omega Bio-tek, M4024-00) and quality-checked by PicoGreen (ThermoFisher) and NanoDrop. The library was prepared by the KAPA HyperPrep for WGS (Roche, KR0961) and sequenced on the Illumina HiSeq X Ten (PE 2 × 150).

**Fig 1:**
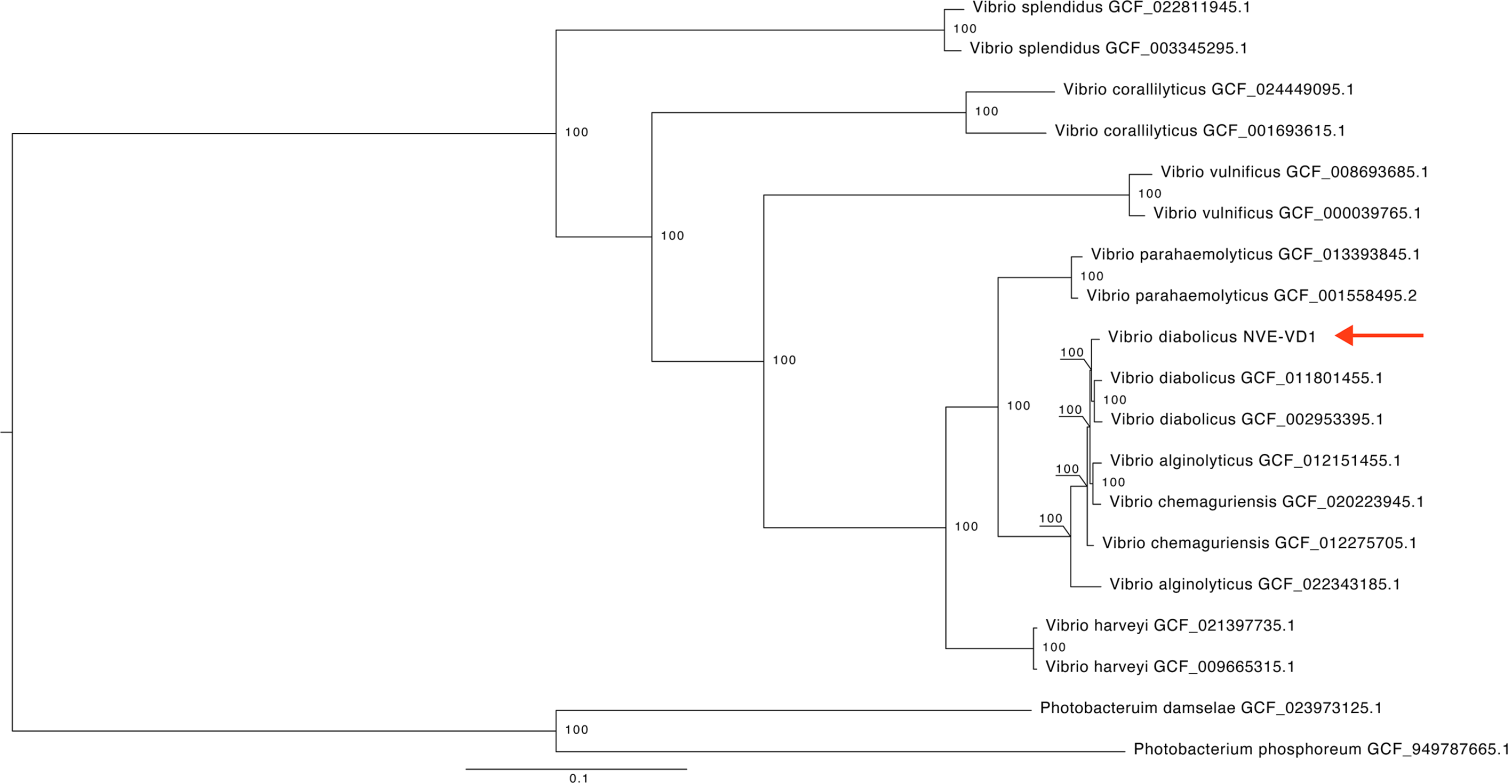
Phylogenetic tree of *Vibrio diabolicus* NVE-VD1 single-copy genes. The single-copy genes of the clade were identified with Orthofinder v2.4.0 and aligned with MUSCLE. The maximum-likelihood tree was constructed in IQTREE2 v2.1.2 and visualized in FigTree v1.4.4.

Paired-end read files were quality-checked with FastQC v0.11.9 (1). A total of 23,441,293 reads were generated from the sequencing run (Table 1). The raw reads were then adapter-clipped and quality-trimmed using Trimmomatic v0.39, which resulted in 16,975,243 remaining reads (2). Next, the trimmed reads were assembled with Unicycler v0.5.0 for *de novo* assembly (3). The final assembly metrics were assessed using Quast v5.0.2 (4) and CheckM v1.2.2 (5) to determine genome completeness and identify any potential contamination. The genome was subsequently annotated with PROKKA v1.14.6, using the options --genus Vibrio and –mincontiglen 500 (6). Next, orthogroups containing 1,309 single-copy genes identified with Orthofinder v2.4.0 were aligned using option -A muscle (7). ModelFinder determined that the best-fit model according to the Bayesian Information Criterion was LG+F+R6 (8). This alignment was used to generate a maximum likelihood phylogenetic tree in IQTREE2 v2.1.2, with 1,000 bootstraps (9). This tree was then visualized in FigTree v1.4.4. Lastly, taxonomic ranks were predicted with NCBI’s PGAP build 6771, using the –taxcheck-only option (10). The *Vibrio* isolate described here was predicted to be *Vibrio diabolicus*, based on the phylogenetic distance of single-copy orthologs (Fig. 1). The constructed maximum-likelihood tree places this isolate in a monophyletic clade with *Vibrio diabolicus*. We designate this isolate *Vibrio diabolicus* strain NVE-VD1. The reference isolate sharing the most genetic similarity with *V. diabolicus* NVE-VD1 was *Vibrio chemaguriensis* with an Average Nucleotide Identity of 98.136% (NCBI accession number ASM1227570).

**TABLE 1:** Genome metrics for *Vibrio diabolicus* NVE-VD1 including sequenced reads, assembly statistics, and genome completeness

	*Vibrio diabolicus*
Raw reads	23,441,293
Trimmed reads	16,975,243
	Unicycler
Statistics without reference	Scaffolds
# contigs	93
Largest contig	1,863,922
Total length	5,117,331
N50	1,302,326
N75	849,246
L50	2
L75	3
GC (%)	44.75
**Mismatches**	
# N’s	575
# N’s per 100 kbp	9.77
CDS	4,541
rRNA	10
tRNA	98
tmRNA	1
**CheckM**	
Completion	100.00
Contamination	3.67

The final assembly was 5,117,331 bp, with a GC content of 44.75%. There were 93 contigs, where the L50 was 2 and N50 was 1,302,326 bp. Interestingly, we found that 309 genes were duplicated, including stress/resistance proteins, secretion system genes, transporter genes, and motility/adhesion genes. These duplications may allow for additional flexibility in the interactions the bacteria have with the host.

## Data Availability

The assembled genome has been deposited to NCBI under the accession number PRJNA1036627. The raw reads were submitted to SRA under the run number SRR26700856.
